# TCW: Transcriptome Computational Workbench

**DOI:** 10.1371/journal.pone.0069401

**Published:** 2013-07-17

**Authors:** Carol Soderlund, William Nelson, Mark Willer, David R. Gang

**Affiliations:** 1 BIO5 Institute, University of Arizona, Tucson, Arizona, United States of America; 2 Washington State University, Pullman, Washington, United States of America; Universidad Miguel Hernández de Elche, Spain

## Abstract

**Background:**

The analysis of transcriptome data involves many steps and various programs, along with organization of large amounts of data and results. Without a methodical approach for storage, analysis and query, the resulting ad hoc analysis can lead to human error, loss of data and results, inefficient use of time, and lack of verifiability, repeatability, and extensibility.

**Methodology:**

The Transcriptome Computational Workbench (TCW) provides Java graphical interfaces for methodical analysis for both single and comparative transcriptome data without the use of a reference genome (e.g. for non-model organisms). The singleTCW interface steps the user through importing transcript sequences (e.g. Illumina) or assembling long sequences (e.g. Sanger, 454, transcripts), annotating the sequences, and performing differential expression analysis using published statistical programs in R. The data, metadata, and results are stored in a MySQL database. The multiTCW interface builds a comparison database by importing sequence and annotation from one or more single TCW databases, executes the ESTscan program to translate the sequences into proteins, and then incorporates one or more clusterings, where the clustering options are to execute the orthoMCL program, compute transitive closure, or import clusters. Both singleTCW and multiTCW allow extensive query and display of the results, where singleTCW displays the alignment of annotation hits to transcript sequences, and multiTCW displays multiple transcript alignments with MUSCLE or pairwise alignments. The query programs can be executed on the desktop for fastest analysis, or from the web for sharing the results.

**Conclusion:**

It is now affordable to buy a multi-processor machine, and easy to install Java and MySQL. By simply downloading the TCW, the user can interactively analyze, query and view their data. The TCW allows in-depth data mining of the results, which can lead to a better understanding of the transcriptome. TCW is freely available from www.agcol.arizona.edu/software/tcw.

## Introduction

With next generation sequencing (NGS), the amount of transcriptome data is increasing rapidly. Typical analyses performed on transcripts are GC-content, open reading frames (ORF), single-nucleotide polymorphisms (SNP), comparisons with protein databases, gene ontology (GO) [1], differential expression (DE), and homology (paralogs and/or orthologs) clustering. Often, publications for transcriptome analysis reference many different programs and perform computations on web sites, which indicates the authors needed to merge the results from the various locations and programs. Most publications do not state what software they used for merging the results, which indicates either that they did not properly reference the software, or they wrote their own scripts and/or used Excel spreadsheets. This causes an ‘ad hoc’ style of analysis that can lead to human error, loss of data and results, inefficient use of time, and lack of verifiability, repeatability and extensibility. Moreover, this approach does not make the data and results easily available on the web in a queryable form for the community.

The Transcriptome Computational Workbench (TCW) aims to make analysis more systematic by consolidating data, analysis and results. Towards this end, TCW contains two manager programs: singleTCW has a graphical interface for building a database of annotated sequences with DE results for a single species, and multiTCW has a graphical interface for building a database of multiple species with comparison results. It uses external programs when appropriate, where most are packaged within the TCW for ease of installation. Except for downloading annotation databases from the web (for which scripts are supplied), the TCW is a web-free program so the user is not dependent on having a good Internet connection or contention with other web users. For large projects, it is beneficial to have a high-end computer, but a 32 CPU 2.4 ghz AMD machine with 128G of RAM and 7TB internal disk space can now be purchased for less than $6000. It is helpful to have a system administrator to configure the machine, but it is now common for biology departments to have such support. To keep installation simple, the TCW uses the common platforms Java, MySQL, and R (optional for differential expression analysis).

Many transcriptome publications show ‘big picture’ results, e.g. a chart of the major GO categories. Though this information is worthwhile, it is equally important to be able to drill down into the data and view exactly what the alignments look like, all the annotation hits (not just the best e-value), etc. Interactive data mining can provide a better understanding of the transcriptome and lead to new and better experiments. The TCW provides this interactive exploratory environment within both singleTCW and multiTCW. A big advantage of using Java is that the project analysis can be run locally for speed, but then results can be made publically available as an applet on the web (albeit, startup time is slower).

The TCW takes as input Sanger reads, 454 reads and/or pre-assembled transcripts (e.g. Illumina) with read counts. It does not perform assembly of high volume short reads as there are many good software programs to perform that task along with computing the read counts (see [2] for a good review). Hence, the TCW can assemble a mix of long reads and transcripts, computing the counts of the reads and integrating the read counts of the transcripts. It will also work directly with a pre-assembled transcript set (i.e. no assembly needed). Though this manuscript is written for transcriptome analysis, the system can also be used for the annotation and DE analysis of shotgun proteomics, i.e. in both cases there are sequences and abundance levels. This would be especially advantageous when joint transcriptome and proteome experiments are performed, as the analysis would be the same for both datasets. To our knowledge, there is no other program that can work with this set of inputs.

The TCW is a combination of pipeline software with a graphical interface and interactive graphics of the results for both single and multiple species. To our knowledge, there is no other software that performs both single and multiple analyses with interactive display. However, there are freely available software programs that have overlapping functionality. As the TCW is specifically written for “project-specific desktop interactive transcriptome analysis”, we compare its features with similar freely available downloadable interactive programs (i.e. not web-based or configurable pipelines).

Both TCW and Blast2GO [3] provide annotation from protein databases and GO assignments along with interactive graphics of the results, but they are otherwise quite different. Using Blast2GO, the user does not need a multiprocessor machine to run the blasts as it uses a cloud server. They do not need to download databases, and the results include InterPro [4] and pathway data. Using TCW, the user can easily configure it to use their annotation databases of choice (protein or nucleotide), it results in an in-house MySQL database of all data and results with differential expression analysis and comparisons across transcriptomes, and it has the ability to provide the results on the web. Both TCW and Blast2GO are written in Java and aim to provide an easy interface for the user. A scenario that would utilize the best of both applications is to use the TCW to output to a file the sequences from the differentially expressed annotated transcripts, and then further analyze the sequences with Blast2GO for pathway, InterPro or other Blast2GO specific features.

An important set of R statistical packages for transcriptomics relates to normalization and DE analysis. The RobiNA software [5] has packaged the edgeR [6], DESeq [7], and EDASeq [8] commands into a Java program, along with executing Bowtie [9] to align the short reads, but does not provide other features of the TCW. The TCW starts processing after the alignment but before normalization and DE analysis. It has packaged the edgeR, DESeq, EDASeq, and DEGseq [10] commands along with the GOseq [11] commands for GO differential expression analysis.

There are a number of programs for the computation of orthologs (e.g. orthoMCL [12] and InParanoid [13]), but they do not have a desktop interactive display. The one exception is OrthoInspector [14], which is a Java package with both a novel algorithm and graphics. In contrast, the TCW does not have its own clustering algorithm except for a simple transitive closure based on BLAST e-values, which provides a way to view the most similar sequences regardless of inparalogs, outparalogs or orthologs. The aim of TCW is more general purpose, as it takes as input any type of clusters (homologous, co-expression, etc.) for combined query and display. OrthoMCL is one of the most widely used ortholog clustering programs, which works for multiple species input. However, it does not provide a way to view the results and assumes knowledge of the Unix command-line environment as it has many steps to perform. Therefore, the TCW has packaged this program for orthologous clustering to make it easier for the user to compute and display orthoMCL clusters.

The TCW provides query and display of clusters, but not the highly graphical displays of CytoScape and its plugins. It provides query and display of read counts (raw or RPKM) along with DE values, but it does not provide the extensive analysis and display of programs such as MeV [15]. Instead, the TCW provides various forms of output that can be used as input into these Java desktop programs in order that a biology laboratory can create an “environment” of interactive graphical software to rigorously explore their data and results.

In genomics, there is a range of software for web-based computing where some sites provide full service (i.e. hardware, software, storage, backups), such as Galaxy [16] and iPlant [17], or just the hardware and software such as ArrayExpressHTS [18] and MyRNA [19]. This is part of the wider trend towards ‘cloud computing’ or ‘software as a service’. The advantages of web-based computing are that the lab does not need to purchase a high-end machine, install software, or (in some cases) provide backups and storage. The advantages of local computing on a high-end machine (preferably that belongs to the project) are that all data is in one place, the lab can more easily collaborate with computational scientists to experiment with new software approaches, and interactive local programs (e.g. written in Java) can be much faster for exploring the data. Local computation also reduces privacy concerns and complications related to data transfer, task-sharing, and detection and re-running of failed processes; moreover, with the steadily-falling price of standalone hardware, the cost is not necessarily higher than a cloud solution. As further discussed in the Discussion, we contend that genomics needs a mix of cloud based and local computing for years to come to give biologists maximal flexibility for exploratory research.

Much of the TCW software has grown out of years of collaborating with biologists on transcriptome analysis, where the original program was referred to as PAVE (Program for Assembling and Viewing ESTS) [20]. Due to its evolution to next-gen sequencing support and comparative analysis, the name was changed to Transcriptome Computational Workbench. This upgraded software was developed for a plant-based project (www.plantrhizome.org), which is sequencing the transcriptomes of rhizomes and other tissues from over a dozen plants to determine what genes distinguish the rhizome from other tissues. None of these plants have sequenced genomes, and only a few have closely related sequenced genomes. Due to the amount of data being generated, it was important to develop a methodical and easy system to use, hence, the TCW. Results are published elsewhere (e.g. He et al. [21]), but timing results will be included here from the rhizome project.

## Methods

TCW is composed of five graphical interfaces: runSingleTCW for building the singleTCW database, runDE for adding DE results to the database, viewSingleTCW for query and display of the results, runMultiTCW for building a comparison database, and viewMultiTCW for query and display of the results.

### Build Single TCW Database (runSingleTCW)

#### Input

**Figure 1:**
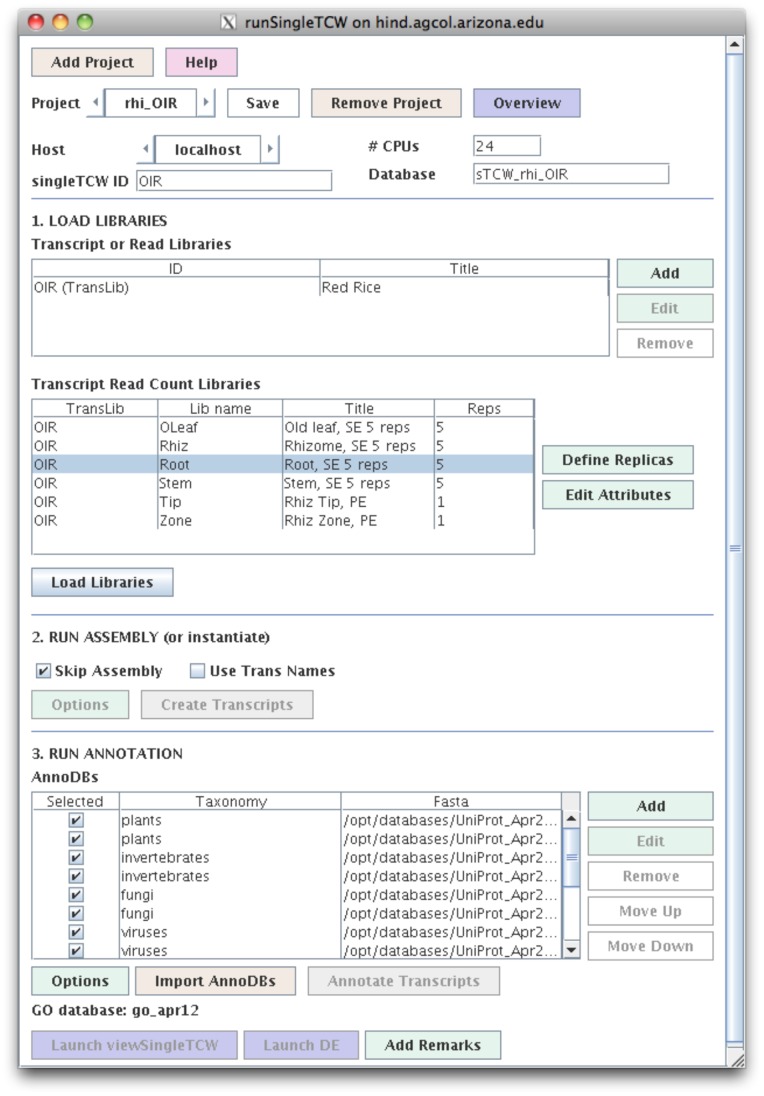
The runSingleTCW interface. This shows the configuration for building the red rice singleTCW database. The “Add” button in the LOAD LIBRARIES section opens a window (not shown) for defining the location of the sequence file and read count files; when the window is closed, information about the read count files is automatically entered into the “Transcript Read Count Libraries” table. The “Define Replicas” button opens a window to allow the user to define the replicas, which results in an updated “Transcript Read Count Libraries” table. In this case, there were 6 tissues types where the first 4 have 5 replicas each. As there was just one pre-assembled transcript data set, it was instantiated without assembly. The 13 annotation databases along with the GO database were identified in the third step.

The only required input is one or more FASTA files of sequences. This program works for any of the following scenarios: (1) assemble Sanger and/or 454 reads, which generates consensus sequences and read counts; (2) import transcripts (e.g. assembled Illumina consensus sequences or gene models) with read counts, which may have replicas for DE analysis; (3) assemble multiple transcript libraries, maintaining the read counts; (4) assemble Sanger and/or 454 reads with transcripts, maintaining the transcript read counts and computing the Sanger and/or 454 read counts; and (5) import protein sequences with abundances from proteomic experiments. We have used the TCW for all five of these scenarios. The manager provides the following steps (see Figure 1):

#### Step 1. Load libraries

The user defines one or more read or transcript files, with optional quality files. For transcripts, they may also define the read count files (multiple files or one delimited file). Replicas (if any) are grouped per library, with values summed for display and used separately for DE analysis. The user defines a short column heading for each library to be used in viewSingleTCW. The user also enters the metadata title, species, cultivar, strain, tissue, treatment, sequencing lab, and year to be displayed on the viewSingleTCW overview page.

#### Step 2. Assemble or instantiate

As detailed in Soderlund et al. [20], the assembly algorithm performs multiple iterations of self-blast and assembly using CAP3 [22]. It was initially developed for Sanger sequences, where mate pairs are retained in the same contigs. It was then extended for 454 reads, which tend to have a higher abundance of reads compared to Sanger, so the algorithm removes reads contained in another from assembly, and after assembly, they are added back into contigs as ‘buried’ reads. Since the original publication, the code has been ported from Perl to Java, plus the following three features have been added. First, SNPs are computed using a binomial p-value based on the number of confirming reads, the depth of the contig at that base, and the estimated base call error rate. Second, the code was extended for already-assembled reads (e.g. Illumina) by allowing transcripts with their optional read counts to be instantiated (i.e. no assembly is performed) or an assembly may be performed while maintaining input read counts. Third, normalization and differential expression statistics are computed, as discussed below.

#### Step 3. Annotation

The user can define one or more protein or nucleotide databases to blast against, and attach a ‘taxonomy’ identifier to each to be used as a short tag for query on a specific database in viewSingleTCW. If the UniProt [23] taxonomic databases are used, each entry is given a tag to identify the taxonomy (i.e. plant, invertebrate, etc.) and whether is it SwissProt or TrEMBL. Additionally, the GO terms, KEGG [24], EC [25], and Pfam [26] identifiers are extracted from the UniProt ‘.dat’ files and added to the TCW database for query and display. The GO analysis uses the GO tree downloaded from www.geneontology.org to extract the levels and descriptions.

During annotation, the GC content and ORFs are computed. The user also has the option to have a self-blast of the sequences run and all similar pairs identified, i.e. this is a good way to estimate how stringent the assembly was.

### Normalization and Differential Expression Results (runDE)

Step 2 above automatically computes RPKM (Reads Per Kilobase of transcript per Million mapped reads) normalization on the raw values. As Mortazavi et al. [27] observed, two transcripts may have the same actual expression level but the longer transcript will have more reads than the shorter one, falsely giving it a higher apparent expression level; therefore, the length must be taken into account. Step 2 also computes the Poisson-based R statistic [28], which was designed for Sanger EST data and takes into account library size and frequency of the read count in all libraries. Though this should not replace use of a more rigorous statistic (e.g. as provided by the DE R packages), it has the virtues of being computationally inexpensive and testing a whole group at once, rather than performing pairwise comparisons.

With the advent of next-gen sequencing, a number of normalization and DE analysis programs have been written in R to take into account the unique attributes of RNAseq data. The EDASeq package performs (among other functions) normalization that takes into account GC-content biases because GC-rich and GC-poor reads tend to be under-represented, and GC-content effects tend to be lane-specific [8]. The edgeR method uses variability between biological replicas to compute the dispersion parameter for a negative-binomial model, which improves the reliability of the differential expression statistic between two libraries; though edgeR can be used without replicas, they strongly suggest having biological replicas [6]. DEGseq provides several different methods, of which three are implemented in TCW: Fisher’s exact test, likelihood ratio test, and an MA-plot-based method [10]. DESeq [7] also uses the biological replicas to compute the dispersion [29]. GOseq [11] computes DE p-values for GO terms based on enrichment of underlying DE transcripts. There are other R packages for DE analysis (e.g. BaySeq [29]), which are not included within the TCW but we would add upon request, or could be added to the downloadable code.

**Figure 2:**
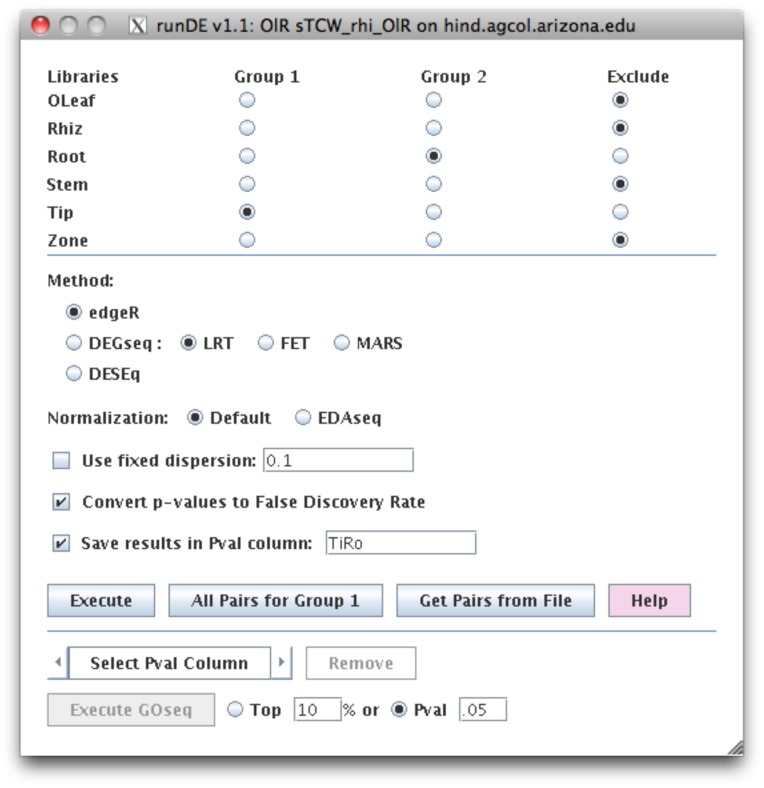
The runDE interface. This shows the setup to compute the differential expression (DE) between tip and root using the edgeR method with default normalization. The results will be written into a database column called “TiRo”. Once all DE values are computed, the “All Pval Columns” can be selected followed by “Execute GOSeq” to compute the corresponding GO differential expression p-values.

To run any of these computations requires some knowledge of R and often requires many commands to be performed. To simplify this analysis for the user, the commands are bundled within runSingleTCW. The user can request default or the EDAseq normalization (see Figure 2), i.e. all three packages have default normalization or can take as input normalized values. Some of these programs provide useful graphing and exploratory functions, therefore, the execution finishes with the R session active at the terminal along with the commands to generate selected graphs, e.g. edgeR provides a scatter plot which can be viewed with “plotBCV(d)”. Columns can be added and removed with results from the different analyses so the user can visually evaluate the results in viewSingleTCW.

**Figure 3:**
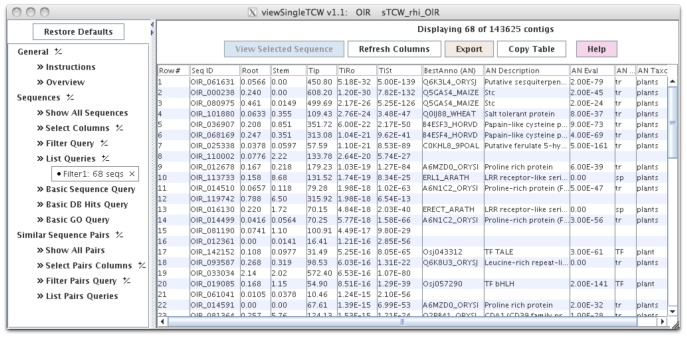
The viewSingleTCW interface. This shows the main table, where the filter was set to show all sequences where tip is differentially expressed compared to root and stem using a 1E-10 cutoff. The columns are: Root, Stem and Tip are the RPKM values; TiRo and TiSt are DE p-values between tip-root and tip-stem, respectively; BestAnno (AN) is the best annotation for the sequence (i.e. not containing phrases such as “uncharacterized protein”); and the last four columns are the BestAnno description, BLAST e-value, the DB type (‘tr’ = TrEMBL, ‘sp’ = Swiss-Prot, ‘TF’ = plantTFDB), and the taxonomic type (all these best hits are from plants).

Though the user can select multiple libraries for each of the two groups, the code treats all the sequences in each group as replicas, hence we recommend that the user perform all pairwise comparisons of interest, and then use viewSingleTCW to search for all or any of the p-values that pass the user-supplied cutoff. For example, with the rhizome data, to ask what transcripts are differentially expressed in rhizome tips compared to stem and root, p-values are computed for each pair (tip-root and tip-stem), then the query requests all transcripts that have a p-values less than a user-supplied value for both columns of p-values (results in Figure 3).

### Single TCW Query and Display (viewSingleTCW)

As shown in Figure 3, the graphical interface is similar to the BioMart [30] approach, where the user selects the columns (attributes) to view and the filters for searches, where the salient filters are on RPKM values, DE p-values, fold changes, ORFs, and attributes of the sequences such as length. The search results in a table of sequence identifiers and associated information, where the table is “full-featured”, i.e. columns can be moved and multiple columns sorted.

A row in the query result table can be selected to view details of a sequence, e.g. alignment of reads to the consensus (assembled only), alignment of hits from the annotation databases, listing of hits, the GO tree of the hits, and other information about the sequence such as ORF and GC content. The alignment of the annotation hits to the sequences uses dynamic programming, and allows the user to see exactly how the hits aligned. The sequence can automatically be sent to the UniProt website to be aligned to all current sequences in that database.

A Basic DB hit query allows search by hit identifier, hit description, or attributes; the results are shown in a table of hit identifiers (i.e. UniProt IDs), with the number of sequences that contained the hit along with the associated UniProt information. The user can drill down by selecting a row and view all the associated sequences. A Basic GO query is very similar; filters include GO number and description keywords, along with DE p-values from GOseq, and the “level:” of the GO (an approximate concept as the GO is not structured as a tree [31]).

### Build multiTCW Database (runMultiTCW)

#### Input

**Figure 4:**
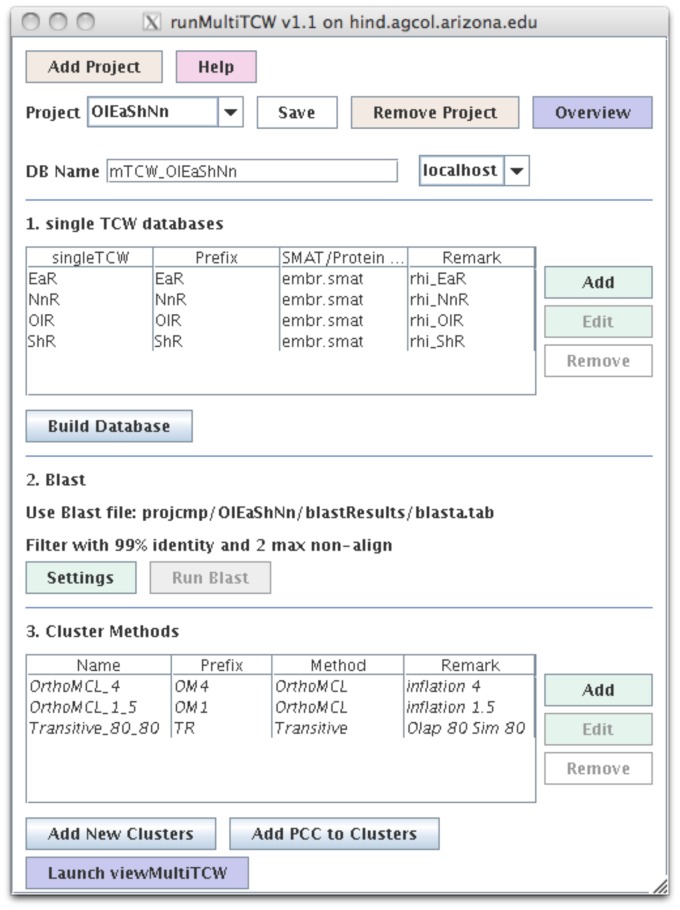
The runMultiTCW interface. This shows the configuration for building a comparison multiTCW database of 4 rhizome singleTCW databases. Three cluster methods were performed: orthoMCL with inflation 1.5, orthoMCL with inflation 4, and transitive with 80% similarity and 80% overlap. The italicized methods indicate they have been added to the database; new clusters can be added and will not be italicized until processed.

One or more databases built with singleTCW. As shown in Figure 4, there are three steps to building a multiTCW database:

#### 1. Import and extract coding sequence

The user selects one or more singleTCW databases. For each database, the user selects the SMAT file for ESTscan [32] to apply to the sequences (the SMAT file is created from related gene Genbank files using an ESTscan supplied script). ESTscan is executed on all sequences and the resulting proteins loaded into the database. If the singleTCW contains protein sequences, then ESTscan is not run. All annotation hits from the singleTCWs are added to the multiTCW database.

#### 2. Run BLAST and filter

The user can supply a blast file of the self-blast of the combined FASTA file produced by step 1, or they can request that the TCW execute the blast. As some of the sequences from a given database can be almost identical (depending on the approach used to create the sequences), the user has the option of removing very similar sequences (based on user supplied parameters of overlap and similarity).

#### 3. Clustering

The user can select one or more clusterings to be performed. For example, they can request orthoMCL with inflation = 4, orthoMCL with inflation = 1.5, transitive closure with overlap cutoff = 80% and similarity = 80%, and upload a file of clusters generated by some other method. This would result in four cluster sets in the database. The annotation that hits the most sequences in a given cluster is assigned to it. Clusters may be removed or added at any time.

### Multi TCW Query and Display (viewMultiTCW)

**Figure 5:**
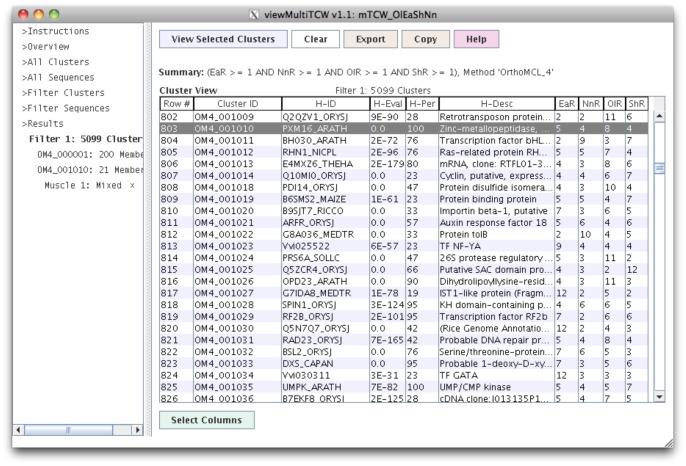
The viewMultiTCW interface. This shows the table of orthoMCL inflation 4.0 clusters where there is at least one sequence from each species. The H-ID and H-Desc columns are the annotation hit that the majority of the sequences have, e.g. for cluster OM4_0001010, 100% have the hit PXM16_ARATH where the best of them has an e-value of 0.0. The columns can be changed by selecting the “Select Columns” at the bottom, which will immediately change the table (i.e. a new search of the database is not required).

The filters allow the user to select a cluster set to view (e.g. orthoMCL with inflation 4), select clusters that have a specific species composition (e.g. only clusters with red rice and horsetail sequences), or select based on RPKM composition. Selecting a cluster results in a full-featured table of the sequences contained in the cluster, as shown in Figure 5. The sequences can be aligned with MUSCLE [33] or the sequences can be pairwise aligned. In both cases, the alignments can be viewed in graphical or sequence detail. A sequence can be selected to view more detail, e.g. all of its annotation hits. The columns of the sequence and cluster tables work differently from singleTCW, as they can be interactively added and removed from the table without a new query of the database.

### The TCW Package

A major objective of the TCW is ease of use, starting with installation. Towards this end, everything is kept as simple as possible so that the biologist can download the package and immediately try it on the demo.

#### Platform

The TCW uses the common platforms of Java and MySQL. There are two Perl scripts, one to download all desired UniProt taxonomic databases and extract the sequences and the second to download the GO tree and merge UniProt information with it.

#### External software packages

TCW uses the external programs Blast, CAP3, MUSCLE, ESTscan and orthoMCL; all external software packages except BLAST are included in the package (TCW can use the old BLAST or new BLAST+). In case of version updates, as long as there is not a format change, the user can easily replace external binaries. For ESTscan, the user needs to make a SMAT matrix from similar sequences (instructions are provided); all other steps are run by runMultiTCW. The orthoMCL package has many steps to perform including one that creates and uses a MySQL database; all steps are performed by runMultiTCW using the user-supplied value for the inflation parameter. The only drawback of including external programs in the TCW packages is that users may neglect to include their respective references in publications that use software within the TCW, hence, the TCW user agreement requires referencing TCW plus all other programs used for the analysis that are contained in the package.

#### R packages

These cannot be easily included in the package, but can all be easily installed with Bioconductor [34]. The TCW package provides instructions to step the user through the installation, which is only necessary if the user desires the DE calculations.

#### Command line

RunSingleTCW writes a configuration file that is used by the loader, assembler, and annotator executables. These programs can be run from the command line once the configuration file is created.

#### Demo and Help

Three single demos are available, as follows: one with Sanger ESTs plus Illlumina transcripts to assemble, one with transcripts and three libraries with three replica biological samples, and one with transcripts and two libraries with no replicas. These three databases can then be used to create a multiTCW database. All necessary files are contained in the package (e.g. the subset of the UniProt files for annotation) with the exception of those needed for DE and GO analysis, where the R packages would need to be installed and the GO tree downloaded, respectively. All interfaces have a “help” button to provide information about the interface. There is also a tour of the interfaces at http://www.agcol.arizona.edu/software/tcw/tour.

#### Interaction with other programs

ViewSingleTCW outputs multiple types of files from the table of query results: (1) all columns from the table as a CSV file, (2) the sequences from the table as a FASTA file (e.g. used as input to Blast2GO), and (3) the Pearson Correlation Coefficient will be computed for co-expression analysis using the RPKM columns (optionally log2 of the RPKM) in the table. ViewMultiTCW will export the contents of any table.

As a general way to add information into the singleTCW database, the user can simply import a file of ‘seqID <remark>’ rows, which can be viewed and searched on in the ‘Remark’ column.

## Results

Results for the assembler are provided in Soderlund et al. [20]. Results are provided here using a dataset from the rhizome project (He et al. submitted) on timing and memory, as detailed in Table 1. The dataset used is from red rice (*Oryza longistaminata*) with 143,625 contigs and read count files for two paired end libraries plus 4 single-end libraries with 5 replicas each (22 total). The transcripts are annotated against the following 13 databases: plantTFDB [35], 5 taxonomic SwissProt and TrEMBL databases, and a SwissProt and TrEMBL subset of the entire UniProt (i.e. minus the 5 taxonomic and bacteria databases). The overall time required to perform the blasts of these 13 databases was a little over 6 days. Loading them into the TCW database along with the GC/ORF analysis and adding GO annotation took ∼14.5 hours.

**Table 1:** Timing results for building a singleTCW database.

SingleTCW	Time	Remarks
Load transcripts	12 m	143,625 transcripts plus 10 read count files
Instantiate transcripts	5 m	No assembly
Blast	6 d:5 h:00 m	13 databases^1^ using 24 CPUs
Add hits and descriptions	13 h:50 m	Add 2M hits, 0.8M unique
Add GO terms	43 m	Add 4.7 k transcript GOs, 17 k unique GOs
DE with edgeR+FDR	9 m	One pairwise computation
**Total**	6 d:20 h:00 m	MySQL database 2.4G

1 The plantTFDB database, the SwissProt and TrEMBL taxonomic databases for plants, invertebrates, fungi, viruses, vertebrate and a subset of the complete UniProt (i.e. minus the sequences from the bacteria and these 5 taxonomic databases). This is based on the April 2012 UniProt, where this set of databases (plus bacteria) take about 45G disk space.

Table 2 shows the time for building a multi-species database from four rhizome species with the same libraries, and all have approximately the same number of sequences except for one that has a genome sequence, in which case, the gene models were used. The overall time was 7 hours, where the majority of that time was for the self-blast (on 24 CPUs).

**Table 2:** Timing results for building a multiTCW database from 4 singleTCW databases.

MultiTCW	Time	Remarks
Load 4 databases	33 m	431,888 transcripts
ESTscan^1^	8 m	170,311 protein^1^
Run selfblast	5 h:40 m	Using 24 CPUs
Run orthoMCL	18 m	18,184 clusters
Run transitive closure	1 m	7,767 clusters
**Total**	7 h:03 m	MySQL database 1.6G

1 ESTscan does not produce protein sequences for all transcripts.

## Discussion

TCW uses MySQL as its database for a number of reasons. First, it is a common database and a biology laboratory is likely to already have it, which keeps installation simple. Second, orthoMCL uses MySQL, and the GO database is also available as MySQL tables, so there is no need for multiple database platforms. Third, TCW is meant for use by a single project and MySQL can easily handle the amount of data for a single laboratory analysis, i.e. it does not need to scale to sizes envisioned for data centers (e.g. iPlant). For example, a TCW project the size of red rice completely annotated (see Table 1) takes about 2.4G, which is just 0.24% of a terabyte hard drive.

The TCW uses common file formats to keep it simple, plus there is no need for more complicated file formats. Though an objective of the TCW is to reduce the use of spreadsheets, they will continue to be of value. For example, the biologist can export the contents of a table in delimited format, where Excel or a similar program can be used to produce graphs for publication. A second output is the FASTA file, where a filtered set of sequences can be used for analysis in a different program. The third output is the correlation coefficients of the DE values for use in pathway programs. These three file formats support analysis commonly used by individual laboratories.

De novo transcriptome assembly is much more compute-intensive than genome-assisted assembly. In both cases, blasting the transcripts against known protein or nucleotide databases is generally also compute intensive. To de novo assemble and map a project the size of red rice (4133M paired ends for assembly) took the following times (personal communications, Min-Jeong Kim, Gang laboratory): ∼24 h for sequence cleaning with CLC Workbench (www.clcbio.com), 12–24 h for the first round assembly with CLC, ∼1 h for polyA trimming with EMBOSS TrimEST [36], 12–48 h for GapClosing, ∼24 h for the second round of assembly with iAssembler [37], and 1–2 h for mapping a single library. Therefore, it took ∼6 days on modest size computers (12-CPU, 32G Ram, 1.5TB HDD Windows machine for CLC and an 8-CPU, 24G Ram, 2TB HDD Linux machine for all other steps). As shown in Table 1, the annotation blasts took about 7 days on a 24CPU processor (only the blast is parallelized). If a lab has a multi-processor computer, a project the size of red rice can be processed in about 13 days (albeit, UniProt has grown since the Apr-2012 release), and then the team members can use the computer for querying the results. Conversely, the availability of cloud computing will increase, and assembly and blasts are great applications for such computing, where the results can be used as input into TCW.

An important aspect of using a program such as TCW, which could reside on the project machine, is to encourage biologists to have a central location for all files and results. This is in contrast to various members of the team having files on their personal computers, which can cause loss of data. The project machine can contain all of the downloadable programs that the members need, e.g. TCW, MeV, CytoScape, Blast2GO. Since many bioinformatics tools run most conveniently on Linux, it is helpful if the team members know basic Unix commands, but these really are simple and can be learned in an hour or two. Users can log in directly from MACs, or from PCs using approaches such as VNC (Virtual Network Computing, www.realvnc.com). Using this approach, the team members have a shared environment with highly interactive graphics, providing a methodical approach for analysis.

As a final word, in the past, many software programs required a high level of computer expertise to run along with the need to read pages of documentation to figure out what to do. The next-gen software programs provide, in general, easy one-step download and execution. For example, MAUVE [38], SyMAP [39], MeV, CytoScape, and TCW are easy to install Java programs, and Blast2GO and BioLayout [40] are webstart Java programs. All of these programs have interactive graphics for analysis and for viewing the results. It would provide a powerful dynamic approach for large-scale data and analysis of the 21^st^ century to have this highly interactive environment working for laboratory projects, along with cloud computing for the large-scale processing and on-line databases to extract information.
